# Antisense Gene Silencing: Therapy for Neurodegenerative Disorders?

**DOI:** 10.3390/genes4030457

**Published:** 2013-09-10

**Authors:** Troels T. Nielsen, Jørgen E. Nielsen

**Affiliations:** 1 Danish Dementia Research Centre, Neurogenetics Clinic, Department of Neurology, Section 6702, Rigshospitalet, Copenhagen University Hospital, Blegdamsvej 9, DK-2100, Copenhagen Ø, Denmark; 2 Department of Cellular and Molecular Medicine, Section of Neurogenetics, The Panum Institute, University of Copenhagen, Blegdamsvej 3, DK-2200 Copenhagen N, Denmark

**Keywords:** RNA interference, neurodegenerative disorders, CNS

## Abstract

Since the first reports that double-stranded RNAs can efficiently silence gene expression in *C. elegans*, the technology of RNA interference (RNAi) has been intensively exploited as an experimental tool to study gene function. With the subsequent discovery that RNAi could also be applied to mammalian cells, the technology of RNAi expanded from being a valuable experimental tool to being an applicable method for gene-specific therapeutic regulation, and much effort has been put into further refinement of the technique. This review will focus on how RNAi has developed over the years and how the technique is exploited in a pre-clinical and clinical perspective in relation to neurodegenerative disorders.

## 1. Introduction

Gene regulation is of major importance in cellular development, differentiation and homeostasis, and studying gene regulation has been an important field of science for decades. It has become clear that erroneous gene regulation or expression of mutant forms of a variety of genes can be the cause of developmental defects as well as early and late onset diseases including cancer, diabetes and neurodegenerative disorders. The discovery by Fire and colleagues in 1998 that double-stranded RNA could efficiently silence gene expression in *C. elegans* [1] and the subsequent discovery that gene silencing was mediated by 21–22 nt long RNAs [2], was the start of RNA interference (RNAi). In 2001 it became clear that RNAi was possible also in mammalian cells [3], which expanded the use of RNAi from being a valuable experimental tool to study gene function to being a possible therapeutic strategy to suppress expression of disease causing genes. This field of science has since evolved enormously and as of today there are more than 150 approved, ongoing or completed clinical trials using RNAi or other antisense therapy primarily to treat cancers [4]. Furthermore, numerous preclinical trials have been conducted for the treatment of a range of neurodegenerative disorders using antisense therapy, which has provided hope that such therapy can be used in a not too distant future [5,6,7,8].

## 2. Origin of Antisense Molecules

Antisense mediated gene silencing refers to the post-transcriptional silencing of genes using small sequence specific (anti-sense) molecules that through complementary base pairing suppress translation or direct degradation of specific target mRNAs. In general terms, two different pathways for antisense mediated silencing exist, namely silencing directed by RNA molecules (hence termed RNAi) and silencing directed by other oligonucleotides e.g., DNA, locked nucleic acids (LNA) or peptide nucleic acids (PNA). Only RNA mediated gene silencing will be reviewed here.

### 2.1. RNA Interference

**Figure 1:**
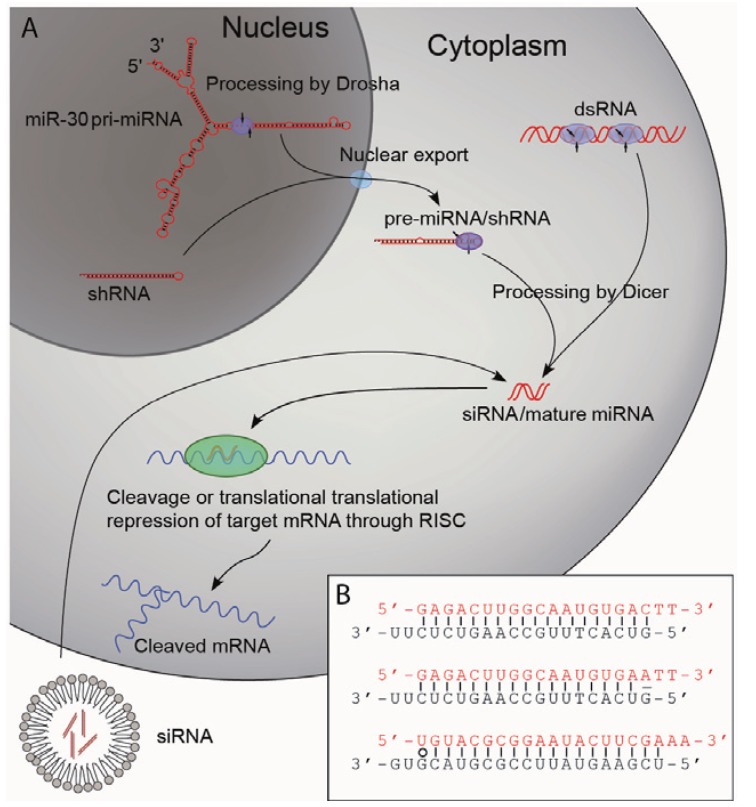
Pathways of RNAi.

The discovery of short RNA duplexes as the mediators of the sequence specific gene silencing led to the elucidation of a general mechanism by which RNAi imposes its effect, namely through a series of cellular events in response to dsRNAs. It was shown that long dsRNAs are cleaved by a cytoplasmic protein called Dicer to produce small interfering RNAs (siRNAs) typically consisting of two 21-nucleotide single stranded RNAs forming a 19 bp duplex with 2-nucleotide (nt) 3' overhangs [9,10,11]. The siRNAs are subsequently loaded into a protein complex termed the RNA induced silencing complex (RISC) in which one strand (the passenger strand) of the siRNA is displaced. The remaining strand (the guiding strand) guides RISC to target mRNA complementary to the guiding strand for endonucleolytic cleavage or translational repression (Figure 1A) [2,9]. The description of this novel pathway led to the discovery of a new group of non-coding RNA molecules, the microRNAs (miRNA). Although small RNA transcripts of approximately 22 and 61 bp complementary to the 3' untranslated region (UTR) of the *lin-14* gene of *C. elegans* were identified in the early 1990s [12], it was not until the early 2000s that such transcripts were recognized as part of an individual group of RNAs with important biological functions—the miRNAs. It became evident that miRNAs are important post-transcriptional regulators of gene expression and that their functions are highly conserved in plants and animals [13]. Today, more than 1,000 miRNAs have been identified in humans [14]. They are transcribed from both introns and exons by polymerase II promoters (transcripts are termed pri-miRNA) and are expressed in a highly regulated temporo-spatial manner. The pri-miRNAs are processed in the nucleus by the endonuclease Drosha to form shorter stem-loop-structures of approximately 70 bp in length (pre-miRNAs) [15,16]. The pre-miRNAs are exported from the nucleus and processed by another endonuclease, Dicer, to form the mature miRNA that consists of 22 nt RNA molecules forming 20 nt RNA duplexes with 2 nt 3' overhangs [16,17,18]. As in the case of the siRNAs the mature miRNAs are loaded into RISC thereby targeting specific mRNAs [19], and siRNAs or dsRNAs thus enter the endogenous RNA processing machinery and share the cellular mechanisms of action with miRNAs.

(**A**) Schematic representation of RNAi using dsRNAs, siRNAs, shRNAs or miRNAs. dsRNAs and siRNA can be introduced by transfection. dsRNAs are cleaved by the cytoplasmic protein Dicer to yield siRNAs. shRNAs are normally transcribed from Pol III promoters (plasmid or viral vector delivery) and are also processed by Dicer yielding siRNAs. Naturally occurring pri-miRNAs are transcribed by Pol II promoters and are processed by the nuclear protein Drosha resulting in pre-miRNAs that after being exported to the cytoplasm are processed by Dicer to form the mature miRNAs. The mature miRNAs or the siRNAs are loaded into the RNA induced silencing complex (RISC) that binds the target mRNA. Naturally occurring miRNAs have only partial homology to target mRNA and bind so-called seed sequences of 6–8 nucleotides in the 3' UTR of target mRNA repressing their translation. siRNAs and shRNAs are designed with 100% homology to the target and direct RISC mediated degradation of the target mRNA. Artificial miRNAs can be produced by exchanging the stem structure giving rise to the mature miRNA with a sequence targeting a transcript of interest. By designing this stem structure with 100% homology to 22 nucleotides of the target mRNA, such artificial miRNA can direct degradation of target mRNA. (**B**) Illustration of the asymmetry of siRNAs. In the upper siRNA the thermodynamic stability of the 5'-ends of both strands is the same, which results in loading of both strands into RISC as the guide strand with roughly the same efficiency. In the middle siRNA a mismatch in the 5'-end of the black strand has been introduced resulting in loading of this strand into RISC almost exclusively. In the bottom siRNA a thermodynamically unstable wobble base pair (marked with o) has been introduced in the 5'-end of the red strand resulting in loading of this strand into RISC almost exclusively. Sequences from Schwarz *et al* [20].

Target recognition of siRNAs and miRNAs is complex. In general, it is believed that siRNAs with complete homology to their target mRNA will bind specifically and promote degradation of the mRNA. However, miRNAs exert their regulatory function by binding to the 3' UTR of mRNAs with only partial homology mediating translational repression rather than mRNA degradation, although recent work has demonstrated that such transcripts are eventually transported to cellular processing bodies and degraded [21,22]. Each miRNA therefore has numerous targets within the transcriptome, and likewise, siRNAs will have numerous target sites with only partial complementarity. Careful design of siRNAs is therefore necessary to minimize off-target effects. Several factors contribute to the specificity of siRNAs and miRNAs. One of the most important is the so-called seed sequence: a 6–8 nt long sequence starting at position 2 in the 5' end of the guide strand [23,24]. Although complete complementarity between the seed sequence and the mRNA has been shown to be crucial for knock down efficacy, base pairing in the central part of the miRNAs have also been shown to be sufficient for miRNAs to exert their function in some cases [25]. Another important point for siRNA and miRNA specificity is how different sequences are loaded into RISC. As mentioned, the passenger strands is displaced from RISC leaving the guide strand to direct mRNA targeting implying that proper guide strand selection is of major importance for specificity. Selection of the intended passenger strand as a guide strand is therefore highly likely not only to abolish silencing efficacy on the intended target but also to confer potent silencing of unintended transcripts. This was elegantly shown by Schwarz and colleagues [20], who showed that the thermodynamic properties of the RNA duplex is a strong determinant for guide strand selection. It was shown that the strand having the weaker binding to the opposing strand in its 5' end is eligible to be loaded into RISC as the guide strand [20]—a discovery that is referred to as the rule of asymmetry. This has since been exploited in the design of siRNAs, where duplexes now are designed asymmetrically to favour optimal loading of the guide strand into RISC. This has been achieved by placement of the siRNA in positions where the targeted sequence has GC-base pairs and AT-base pairs in their 5' end and 3' end, respectively, or by introducing thermodynamically less stable wobble base pairs or actual mismatches in the 5' end of the guide strand (Figure 1B) [20,26,27]. In addition to the careful design of the siRNA duplex in order to avoid passenger strand loading, the seed sequence of the siRNA should be given a bit more consideration. It has been elegantly shown by Boudreau and colleagues that the degree of off-targeting is closely correlated to the number of seed sequence matches in the 3' UTR of the entire transcriptome. The seed sequence should therefore not only completely match the target mRNA of interest, it should also be as infrequent as possible in the 3' UTR of the transcriptome [26]. These data demonstrate the complexity of gene regulation by small RNAs, and underscore the need for thorough screening both *in silico* and in experimental settings of siRNA candidates to evaluate the quality of both efficacy and specificity of a given siRNA.

Several tools for designing siRNAs, shRNAs and artificial miRNAs exist online. However, for the most part design algorithms and exactly which rules are used and how these are applied in the design and rating of different sequences remains proprietary information of the companies providing the services. Therefore, design of siRNA should be followed by thorough experimental validation to avoid false positive sequences and to determine the actual efficacy of the sequences and their off-targeting profile.

### 2.2. Biogenesis of Small RNAs

Initially, dsRNA and siRNAs were synthesized and injected directly into the cells of *C. elegans* and later siRNAs were introduced into mammalian cells by transfections [1,3]. It is evident that direct injection into individual cells is a suitable method neither for experimental setups where large numbers of cells need to be targeted nor for gene therapeutic applications. On the contrary, transfection of siRNA using various transfection agents (*i.e.*, oligofectamine) has in experimental settings been widely used, but this method offers only transient suppression of gene expression. This constrain was circumvented by Brummelkamp and colleagues in 2002 [28] by the design of a plasmid vector expressing a short hairpin RNA (shRNA) from the H1-RNA promoter. The H1 promoter belongs to the group of polymerase III promoters that normally transcribe transfer RNAs and ribosomal RNAs. The H1 promoter is characterized by having a well-defined start of transcription and it produces a transcript lacking a poly-adenosine tail and hence, it is capable of producing an RNA transcript in which the ends resemble the ends of synthetically produced siRNA [28]. By designing a gene specific insert of 19 nucleotides that is separated from a complementary sequence of the same length by a suitable spacer sequence, it is possible to achieve a stem-loop structure that upon processing by Dicer will give RNA duplexes similar to that of synthetic siRNAs (Figure 1A). This allows for continuous production of siRNAs and consequently, for the possibility of stable transfectants and long-term gene knock down. The production of siRNAs from promoters active in mammalian cells offers, in addition, the possibility of expressing siRNA from viral vectors whereby cells difficult to transfect can be targeted [29,30,31]. Furthermore, it opens up for several gene therapeutic applications and efficient *in vivo* delivery of shRNA [5,29].

Although vectors expressing shRNA from polymerase III promoters seem good candidates for therapeutic applications, a number of issues have to be considered. Firstly, experimental evidence indicates that shRNA expressed from polymerase III promoters can cause saturation of the endogenous RNA processing machinery by saturating the export of RNA hairpins from the nucleus and by saturating the argonaute proteins of RISC [32,33]. This has led to cytotoxicity and tissue damage that has proven to reduce efficacy of long term treatment or to cause premature death in a mouse model of Hepatitis B [32,34]. Furthermore, polymerase III promoters express ubiquitously and constitutively making targeted and regulated expression difficult. While a few reports exist describing that shRNA can be driven by polymerase II promoters (such as the CMV promoter or cell specific promoters) [5,35,36], a strict requirement for transcriptional initiation and termination seems to persist [5,36]. This is likely to make the general use of polymerase II promoters difficult, since such promoters have to be very well characterized with regard to transcriptional start and termination sites in order to be able to transcribe functional shRNAs.

However, shRNAs have recently been embedded into a miRNA context making it possible to achieve siRNA transcribed from polymerase II promoters in broader terms [37,38]. Since endogenous miRNAs are transcribed by a variety of polymerase II promoters, synthetic or artificial miRNAs have been designed by exchanging the specific stem structure giving rise to the mature miRNA of the pri-miRNA transcript with a sequence targeting a mRNA of interest [38,39]. In this way, the endogenous miRNA processing machinery is exploited to achieve functional siRNAs transcribed from polymerase II promoters. Such artificial miRNA designs present several advantages over shRNA vectors. Firstly, a comparative study of vectors expressing shRNAs and artificial miRNAs has shown no toxic effects of the artificial miRNA-based vectors possibly due to a lower level of anti-sense RNA generated by these vectors [34]. Secondly, reporter genes can be incorporated into the artificial miRNA transcript making it possible to easily track smiRNA expression to individual cells [38]. Finally, the promoter can be exchanged with a variety of different promoters without the need for optimizing the transcriptional initiation site thereby offering the possibility to use cell specific promoters to target siRNA expression to specific cell types *in vivo* [40]. However, although the miRNA embedded shRNAs seem superior to traditional shRNA in terms of expression and toxicity profiles, thorough design of the antisense sequence is still necessary in order to avoid toxicity or other adverse effects caused by off-target silencing.

## 3. Delivery of Small RNAs

Although the technique of antisense mediated gene silencing holds great promise as a therapy against a range of disorders, the issue of non-toxic and efficient delivery of the siRNAs still presents as the greatest barrier for RNAi to reach the clinic in broader terms. For delivery of siRNAs or antisense oligos certain requirements have to be met. Firstly, delivery has to be efficient enough to target the required number of cells to obtain efficacy of treatment. Secondly, the route of administration has to be feasible, especially if prolonged knock down effect is required and re-administration is a necessity, e.g., for non-vector mediated siRNA delivery to chronic disorders. Thirdly, delivery has to be non-toxic and non-immunogenic to avoid adverse effects in this regard. Much effort has been put into the development of systems that fulfil these criteria, some of which will be outlined here.

### 3.1. Non-Viral Delivery

Delivery of siRNAs is most often done by packaging into carrier systems that allow the negatively charged RNA molecules to penetrate the cellular membranes. Furthermore, such carrier systems protect the RNA from the rapid degradation that takes place in serum upon systemic delivery of siRNA as well as the excretion occurring through the kidney. Carrier systems are most often based on unilamellar or multilamellar liposomes in which the siRNAs are contained in a hydrophilic core. The physiochemical properties of the liposomes can be optimized in various ways to enhance delivery efficacy and prolong systemic stability by modification of the lipids with different compounds, e.g., polyethylenglycol [41]. Furthermore, targeted cellular uptake can be obtained by conjugating various molecules to lipid compounds of the liposomes e.g., antibodies or ligands specific for certain cellular receptors [41]. Recently, other lipid-like substances (*i.e.*, lipidoids) have been used with some success *in vitro* and are currently under pre-clinical investigation [42]. Another possible route for introducing siRNAs into cells is by using cationic polymers. These are large linear or branched molecules (e.g., cyclodextrin or polyethyleneimine) that efficiently bind nucleic acids. They are readily taken up by endocytosis and their cargo has been shown to escape the endosomal pathway releasing the siRNA into the cytosol of the cell [43]. Although cellular uptake of such nanoparticles occurs through the endosomal pathway targeted delivery to tumours has been shown using cationic polymers by attachment of targeting ligands to the polymer particles [44]. Finally, naked siRNA has in some instances been used successfully for knock down by conjugating the sense strand of the siRNA to, for example, cholesterol, which has favoured siRNA uptake in neurons and hepatocytes [45,46].

### 3.2. Viral Delivery

As briefly mentioned above, the development of systems in which the small RNA molecules are transcribed from promoters active in mammalian cells has paved the way for delivering small RNAs using viral vectors. Viral vectors take advantage of the ability of viruses to transfer their genetic material for efficient replication. When basing a gene transfer vector on a virus, some of which cause serious or fatal diseases in humans, several precautions have to be taken in order to make the vector systems safe, and the strategy to obtain safe viral vectors is based on separation of the viral components [47,48]. The *cis*-elements necessary for packaging (formation of viral particles) are kept in a transfer vector plasmid, whereas *trans*-elements that code for proteins necessary for virion formation are deleted and provided on one or more helper plasmid lacking all *cis*-elements. By co-transfection of a packaging cell line with these plasmids all viral proteins necessary for the production of viral particles will be expressed from the helper plasmids, whereas the viral genome will be produced from the transfer vector plasmid, whereby infectious viral particles are assembled. The resulting recombinant viral particles will be able to efficiently infect target cells and transfer genetic material, but will be unable to express viral proteins and hence, comprise a dead end where no further viral particles can be produced (referred to as replication incompetent viral vectors).

Several different viruses have been used as delivery vehicles, and based on the native properties of the viruses each of these can be used for specific applications to fulfil a specific need for gene transfer. Some of the most widely used vector systems will be discussed here (see Table 1 for an overview).

**Table 1:** Overview of vector types commonly used in preclinical trials.

Vector	Retrovirus	Lentivirus	HSV	ssAAV, scAAV	Adenovirus
**Genome**	RNA	RNA	DNA	DNA	dsRNA
**Cloning capacity**	8–10 kb	8–10 kb	150 kb	<5 kb, 2.2 kb	Up to 35 kb
**Pseudotype/serotype**	VSV-G LCMV-G Ebola *etc.*	VSV-G LCMV-G RV-G RB-G MV-G Ebola *etc.*	Mainly HSV-1	1–12, Chimeric and engineered	>50 naturally occurring. Type 2 and 5 used for vectors
**Immuno-genecity**	Low	Low	Highly	Mild	Highly
**Pre-existing immunity**	Limited	Limited	Yes	Limited	Yes
**Transduces non-dividing cells**	No	Yes	Yes	Yes	Yes
**Insertion into chromatin**	Yes	Yes	No (Episomal)	Yes/No (Episomal/integrated)	No (Episomal)
**References**	[49,50,51]	[49,52,53,54,55]	[56]	[57,58,59,60,61,62,63,64,65]	[66]

Retroviruses (e.g., Murine leukemia virus) are RNA viruses that are characterized by having two identical copies of a single stranded RNA genome (pseudodiploid) and upon infection the viral genome is reverse transcribed and incorporated into the chromosomes of the infected cells [47]. A subtype of retroviruses is the lentiviruses (e.g., HIV) that have been extensively used for gene transfer. Basing viral vector systems on a lethal virus such as HIV might seem unattractive, but lentiviruses carry characteristics that from a gene transfer point of view are very favourable. First of all, like other retroviruses, lentiviruses infect target cells chronically by integrating their genome into the chromosomes of the host cell, which will make it possible to obtain long-term expression after a single gene transfer event [67]. Secondly, in contrast to simple retroviruses they are able to infect non-dividing and post-mitotic cells such as terminally differentiated neurons, which makes them ideal candidates for gene transfer to the brain [68]. Finally, the envelope protein of vectors based on both simple retroviruses and lentiviruses can be changed to envelopes of other viruses, whereby the cellular tropism of the viral vector can be altered. This is referred to as pseudotyping [69] and it has been used to broaden the tropism of vectors to include target cells not normally infected by the native form of the virus. An ever increasing number of envelope proteins have been used for pseudotyping, and a complete review of all of these is beyond the scope of this review, but a few envelopes will be discussed here (for a more comprehensive review see [52]). One of the most widely used envelopes is the one based on the glycoprotein of the Vesicular stomatitis virus (VSV-G), which has a broad host range and furthermore confers mechanical stability of the virions allowing for concentration of the viral particles by ultracentrifugation [53]. Other envelopes include glycoproteins from Lymphocytic choriomeningitis virus (LCMV-G), Ross river virus (RRV-G), Mokola virus (MV-G) and Rabies virus (RB-G). Each of them has different properties in terms of cellular preference, toxicity and immunogenicity. LCMV-G and RRV-G has been shown to be less toxic than VSV-G still retaining the broad tropism characterizing VSV-G [49,54,55,70]. However, conflicting results have been reported with regard to the cellular preference of pseudotyped lentiviral vectors [71,72], but this is more likely a matter of the capability of different promoters to express in different cell types rather than a matter of the pseudotyped virions’ ability to infect certain cell types [73]. One example is VSV-G pseudotyped vectors that show robust glial expression when using a promoter active in glial cells although such VSV-G pseudotyped vectors were initially reported to have a strong neuronal preference [73,74]. This later turned out to be caused by expression patterns of the used promoters [72,73]. Furthermore, it has been shown that the envelope protein can influence the axonal transport and in this way also influence transgene expression pattern *in vivo*. This strategy was used to transduce motor neurons of the spinal cord and the brain stem in a mouse model of amyotrophic lateral sclerosis upon RB-G pseudotyped lentiviral vector injection into muscle tissue [75,76]. Besides pseudotyping with envelope proteins from native viruses, engineering of envelope proteins can be used to selectively change the properties of the envelope and in this way alter cell specificity, vector stability, transduction efficiency, resistance to antibodies, *etc.* [77,78,79], which is a promising strategy for tailor made envelope properties.

Adeno-associated viruses (AAV) are DNA viruses with a genome size of around 5 kb, and vectors derived from AAVs, thus having a rather limited cloning capacity of 5 kb or less [57]. Despite the limited cloning capacity, they have gained much interest and are one of the most widely used vector systems for gene delivery to the central nervous systems. One reason for this is the AAV vectors’ safety profile. AAVs have not been associated with disease in humans, which makes them ideal candidates for vector development. Furthermore, the vector genome primarily stays episomal, and the integrating proportion of the vector genomes integrate into a well-defined chromosomal area on chromosome 19 [58,59]. It is therefore believed that AAV vectors confer a favourable safety profile with regard to insertional mutagenesis [80,81], although one study reports possible insertional mutagenesis using AAV vectors in a mouse model [82]. On the other hand, insertional mutagenesis has been reported in several instances to cause leukemia in patients treated for X-linked severe and combined immune deficiency using retroviral vectors [50,51,83]. In addition to the desirable safety profile, AAVs have proved to confer long lasting transgene expression in the CNS and to efficiently transduce both dividing and non-dividing cells [60,61]. AAVs are therefore considered a promising tool for gene therapy to the brain. Several different serotypes of AAVs exist. In 1982, the first recombinant AAV vector was published and this was based on serotype 2 [62]. This showed effective long-term gene transfer to the CNS and was primarily targeting neurons [60,61]. Since then several other serotypes have been found and tested. These show different properties in terms of cellular preference, transduction efficiency and the predisposition to neutralizing antibodies [63,84], but although numerous studies have reported on the properties of the different serotypes some inconsistencies exist. For example injection of AAV1, 2, 5 and 8 vectors into the brain has shown mainly neuronal expression in some studies [85,86,87,88] whereas other studies show astrocytic and oligodendrocytic expression from AAV 1, 5 and 8 [87,89,90]. The inconsistencies have been shown to be caused at least partly by the method used for vector production and purification [90]. Aside from the differences in the cellular expression pattern of the different serotypes differences in the area of transduction has also been reported. While AAV2 transduces a rather limited volume upon direct injection into the brain AAV5 and 8 transduce a relatively large area [91]. Finally, AAV9 has gained much interest due to its ability to cross the blood-brain-barrier allowing for easy transduction of the CNS upon intravenous administration [64,92], and recently, much interest has also been put into the development of chimeric AAV serotypes and synthetic serotypes designed to enhance certain traits for more efficient and targeted delivery using AAVs [63]. For a more comprehensive review of the differences of AAV serotypes see [65].

Viral vectors based on adenovirus and herpex simplex virus have also been used in gene therapeutic settings. These hold much larger packaging capacities (see Table 1), but a significant drawback is their immunogenicity that in 1999 caused the death of a 18 year-old man enrolled in a gene therapy programme due to uncontrollable immunologic reaction towards an adeno virus vector [66]. Since gene silencing cassettes are generally small (shorter than 3 kb), AAVs, retroviral and lentiviral vectors can accommodate sufficient genetic material for this purpose, and adeno and herpex simplex virus based vectors will not be discussed in more detail here.

## 4. Therapeutic Applications of Antisense Technology

The technique of RNAi has provided new means of studying gene function and it has provided hope for treatment of diseases that previously had no treatment options. When the potential of RNAi became evident, the pharmaceutical industry initiated large research programs to exploit the new technology, and the hopes were high for drug development with huge economic potential. However, in 2010 several of the major companies curtailed or even ended their research programs due to lack of clinical progress within the field, but although this setback for RNAi therapy seems hard, some companies still maintain their optimism that RNAi will deliver some clinical success, and these companies have therefore sustained their programs to some extent [93]. However, despite the disappointment in the clinical progress of RNAi and the waning of the industry’s belief in a RNAi gold rush, tremendous preclinical advances have been made with regard to the use of RNAi for therapeutic applications towards CNS disorders, and numerous studies have been published showing great promise in various animal models (for an overview see Table 2).

**Table 2:** Overview of selected studies using RNAi for neurodegenerative disorders.

Disorder	RNAi method	Target	Mechanism	Disease model	References
Huntington’s disease	siRNA AAV-shRNA/miRNA	htt	Removal of toxic protein	Cell culture Transgenic mouse models Monkey	[7,34,94,95,96,97,98,99,100,101]
SCA1	AAV-shRNA	ATXN1	Removal of toxic proteijn	Transgenic mouse model	[102]
SCA3	LV-shRNA AAV-shRNA AAV-miRNA	ATXN3	Removal of toxic protein	Rat model Transgenic mouse models	[6,96,103,104]
SCA6	siRNA miRNA	CACNA1	Removal of toxic protein	Cell culture	[105]
Parkinson’s disease	siRNA LV-shRNA AAV-shRNA	α-synuclein LRRK2 GAD67	Removal of toxic protein Modulation of neuronal transmission	Cell culture Mouse model Rat model	[106,107,108,109,110,111]
ALS	siRNA shRNA LV-shRNA Mouse transgenesis, shRNA	SOD1	Removal of toxic protein	Cell culture Mouse model Transgenic mouse models	[112,113,114,115,116]
Alzheimer’s disease	siRNA shRNA LV-shRNA HSV-shRNA	APP PS1 DMT1 BACE1 CDK5	Removal of toxic protein Indirect modulation of APP expression. Modulation of APP processing. Modulation of Tau phosphorylation.	Cell culture Mouse model Transgenic mouse models	[56,117,118,119,120,121,122,123,124]
Multiple sclerosis	LV-miRNA	Act1	Modulation of interleukin-17 signalling	MS mouse disease model (EAE mouse)	[125]
Prion disease	Mouse transgenesis, shRNA	PrP(C)	Removal of wt protein to avoid conversion to toxic species.	Mouse model	[126]

### 4.1. Monogenic Disorders

The monogenic disorders caused by dominant negative or dominant toxic gain-of function mutations provide delicate targets for antisense therapy, since in theory these diseases can be treated and disease progression stopped by the inhibition of the expression of a single gene. One of the most studied disease entities in this regard is the polyQ diseases and in particular Huntington’s disease (HD).

#### 4.1.1. PolyQ Disorders

HD is an autosomal dominantly inherited fatal neurodegenerative disorder that is caused by the expansion of a trinucleotide CAG repeat in exon 1 of the 67 exon containing huntingtin (*htt*) gene resulting in an abnormally expanded polyglutamine tract in the protein huntingtin (Htt) [127,128]. HD is characterized by a progressive atrophy of brain tissue, in particular of the striatum and cortex. The symptoms and signs of the disease are involuntary movements (chorea, dystonia, grimacing, gesticulation, ataxia *etc.*), psychiatric disturbances and dementia with a typical age of onset of 35 to 50 years (range 2–70 years). The disease gradually worsens until death occurs 15–20 years after onset of symptoms [129,130]. The mechanism that causes disease by the expansion of the polyglutamine tract is uncertain, but it is believed that the mutation leads to a toxic gain-of-function [131,132,133,134] and thus, this disorder has served as target for evaluating RNAi therapy. Several approaches have been used to knock-down *htt* in animal models. In 2002, Xia and colleagues published that AAV mediated expression of a shRNA could efficiently and specifically silence gene expression and furthermore, significantly reduce one of the major pathological hallmarks of HD, namely the aggregation of the polyglutamine elongated Htt, *in vitro* [5]. Several follow up studies later showed that the improvement in pathological parameters of *in vitro* studies was paralleled *in vivo* by rescue of motor function in various transgenic mouse models of HD after injection of shRNA expressing AAVs or siRNAs [94,95].

Several other studies have reported similar therapeutic benefit in transgenic models of other polyglutamine disorders. In particular, the spinocerebellar ataxias (SCAs) have been an area of intense research. The SCAs are like HD characterized by a CAG-repeat expansion in their respective gene that through mostly unknown mechanisms cause cell death of primarily the Purkinje neurons of the cerebellum but also in various other brain regions including the cortex and the brain stem [135]. The general symptoms of the SCAs consist of progressive cerebellar ataxia and diverse extracerebellar symptoms [135]. Since the repeat expansions are believed to be toxic gain-of-function, the rationale of using RNAi for the SCAs is to remove the protein to slow down or stop the cellular degeneration. This has been shown to be a viable approach in several animal models of SCA1 and SCA3 (Machado-Joseph disease), in which protein aggregates, cellular degeneration, thinning of the molecular layer of the cerebellum and motor deficits can be prevented upon silencing of the gene underlying the different forms of SCAs (ATXN1 and ATXN3 for SCA1 and 3, respectively) [6,102,103,104]. Although encouraging, several concerns exist. Firstly, the neurodegenerative disorders are often slowly progressing, late onset disorders in which the pathological mechanisms are ongoing through several decades and knock-down of the disease causing genes therefore needs to be persistent over prolonged periods of time. Long term knock-down has been achieved by viral delivery of shRNA or artificial miRNAs as described above or by infusion of siRNAs or anti-sense oligos [6,8,94,95,102,103]. In principle, viral mediated delivery allows for a life-long intervention that, however, cannot be discontinued if desired, whereas infusion of siRNAs or antisense oligos requires repeated interventions but with the possibility to discontinue treatment. Secondly, since knock-down is needed for prolonged periods of time, knock-down of the wild type allele is problematic, and might result in loss-of-function effects. Although several studies have reported that non-allele specific gene silencing of Htt is well tolerated in animal models [7,34], the time frame of these studies is still limited compared to the time frame in question upon treatment of humans. Therefore, allele specific silencing has been a matter of much interest in recent years and different strategies have been applied to achieve this. One strategy is to target the CAG-repeat, which has been pursued by several groups, and it has shown some selectivity between WT and mutant htt when CTG repeat siRNAs are transfected into fibroblast from HD patients [96]. Furthermore, by introducing mismatches to the CAG repeat at specific positions and by shortening the sense strand of the siRNA duplexes, the selectivity can be increased and the targeting efficacy towards other CAG-repeat transcripts minimized [97,98]. The problem of selectively targeting the expanded CAG-repeat is circumvented in another approach where the siRNAs are directed against single nucleotide polymorphisms (SNPs) present in the 3' UTR of the disease causing transcripts [8,99,100]. This has shown promising results and mapping certain SNPs to certain CAG-repeat lengths has shown that by targeting relatively few SNPs, it will be possible to silence mutant Htt in the majority of patients [101]. This approach could possibly be applied to other disorders as well although a prerequisite for this strategy is the presence of targetable SNPs in the disease causing transcripts.

A different approach for selectively targeting the disease causing transcript has been explored in SCA6 that is caused by a polyQ expansion in the 47th exon of the *CACNA1A* gene (encoding a voltage gated calcium channel) [105]. The CACNA1A transcript is differentially spliced in a manner that results in two isoforms: One that allows translation of exon 47 and the CAG-repeat, and one that has a stop codon in the beginning of the 47th exon and hence, does not include the CAG-repeat upon translation [105]. Upon elongation of the CAG-repeat, preferential splicing occurs in favour of the isoform that in which the CAG-repeat is translated implying that the impact of this deleterious mutation will increase further. However, the splice variant allowing polyQ translation has an additional 5 bp sequence at the exon 46/47 junction that is not present in the transcript translated to the shorter isoform, and this small difference in sequence has been used successfully for specific silencing of the disease causing transcript variant [105].

Allele specific silencing generally applies to other diseases including diseases caused by point mutations, e.g., the autosomal dominant forms of Parkinson’s disease, familial forms of Alzheimer’s disease, amyotrophic lateral sclerosis, and frontotemporal dementia.

#### 4.1.2. Parkinson’s Disease

In Parkinson’s disease (PD) the key pathological finding is the selective degeneration of dopaminergic neurons of substantia nigra pars compacta, which results in several changes of the neural circuitry controlling motor function. The symptoms consist of resting tremor, rigidity, bradykinesia and postural instability [136]. Most cases of PD are sporadic but among the inherited forms are mutations in the gene encoding α-synuclein. The function of α-synuclein is poorly understood, but it has been shown that three different point mutations in α-synuclein as well as duplications and triplications can cause the disease and that α-synuclein can be found in aggregates in the brain of PD patients [137,138]. To this end, it is logical to speculate that reducing the level of expression of α-synuclein or reduce expression of the mutant forms of α-synuclein will be beneficial for inherited forms of PD, and this has been investigated by several groups. It was found that knock-down of α-synuclein is possible both *in vitro* and *in vivo* and that α-synuclein knock-down reduced cellular sensitivity to a neurotoxin (MPTP) known to induce PD [106,107]. Similar proof of principles studies have been conducted that show that allele specific silencing of another mutant gene causing PD, the leucine-rich repeat kinase 2 (LRRK2) gene, can be achieved [108,109]. However, recently it has been shown that non-allele specific knock-down of α-synuclein in the substantia nigra in rat brain is accompanied by loss of tyrosin hydroxylase positive cells (tyrosine hydroxylase is the rate limiting enzyme in the dopamine synthesis in neurons) implying that RNAi directed against α-synuclein might actually induce dopaminergic cell loss in substantia nigra and thus be difficult to apply without toxic effects [110]. This is in contrast to RNAi in HD, where non-allele specific knock-down in reality has not yet shown to be a problem in animal models.

#### 4.1.3. Amyotrophic Lateral Sclerosis

Amyotrophic lateral sclerosis (ALS) is a progressive neurodegenerative disorder involving motor neuron degeneration, as well as skeletal muscle atrophy and paralysis. It has a rapid disease cause and is often fatal within five years from diagnosis. Most cases are sporadic; however, mutations in different genes have proven to cause familial forms of ALS. Among these mutations are mutations in the superoxide dismutase 1 gene (*SOD1*), the TAR binding protein gene (*TARBP*), *C9ORF72* and others [139,140]. All of these are inherited in an autosomal dominant manner and they are regarded as dominant negative or toxic-gain-of function mutations and like other neurodegenerative disorders caused by such mutations, these genes have served as therapeutic targets for RNAi. The most widely studied mutations in ALS are the *SOD1* mutations, and allele specific silencing of *SOD1* has been conducted *in vitro* and in transgenic mouse models of ALS [112,113,114]. However, conflicting results have been reported as to the therapeutic efficacy of *SOD1* knock-down. Some studies report promising effects of knocking-down *SOD1* [113,114], whereas others do not [115,116]. For example, Towne and colleagues have published efficient body wide transduction upon systemic delivery of AAV6 expressing an anti-SOD1 shRNA, but without the expected therapeutic effect [115]. They suggest that although efficient transduction of both muscle cells, motor neurons and glial cells, the percentage of transduced motor neurons was probably too low (<5%) to confer a therapeutic benefit [115]. Furthermore, in a follow up study injection of anti-SOD1 expressing AAV6 was targeted directly to vulnerable motor neuron pools conferring high levels of knock-down in the particular neuronal pools, but once again without the expected therapeutic outcome. The authors speculate that the reason should be sought within the lack of global knock-down or the lack of knock-down in other cell types (astrocytes, microglia or muscle cells) [116]. This fits nicely with the study by Xia and co-workers, since they obtain therapeutic benefit of their shRNA by expressing the antiSOD1-shRNA through generation of transgenic mice lines and subsequent crossing with SOD1 mutant transgenic mice [114]. Hence, they obtain global expression of the antiSOD1-shRNA probably accounting for their observation of phenotypic improvement. In conclusion, these results once again highlight the vulnerable point in gene therapy, namely delivery that persistently is challenged by the complex interplay between physical barriers between different tissues and the different cellular components within a given tissue.

#### 4.1.4. Alzheimer’s Disease and Frontotemporal Lobar Degeneration

Alzheimer’s disease (AD) is the most common cause of dementia and in most cases the disease is of unknown cause. Pathologically, the disease is characterized by gross atrophy of the cerebral cortex including the temporal lobe, parietal lobe and the cingulate gyrus. Pathology furthermore includes accumulation of abnormally folded protein, amyloid β and tau, in so-called extracellular amyloid plaques and intracellular neurofibrillay tangles [141]. Familial forms of AD have been shown to be caused by mutations in the genes encoding amyloid precursor protein (APP), presenilin 1 and 2 (*PS1* and *PS2*) [141]. Frontotemporal lobar degeneration (FTLD) comprises a heterogeneous group of disorders which are all characterised by gross atrophy primarily of the frontal and/or temporal lobes. FTLD generally presents with either personality change, termed behavioural variant frontotemporal dementia (bvFTD) (or simply FTD) or distinct language impairments [142]. The neuropathology of FTLD syndromes is extremely heterogeneous and includes tau pathology in FTLD caused by mutations in the microtubule-associated protein (*MAPT*) gene whereas FTLD caused by mutations in the progranulin (*GRN*) gene causes tau-negative, TDP-43 positive inclusion pathology [139]. The familial forms of AD and FTLD are all dominantly inherited through dominant negative or gain-of-function mechanisms.

Several preclinical studies using RNAi have therefore been conducted to knock-down the mutant genes. For instance, it has been shown that the cellular sensitivity to capsase-3 activation and apoptosis is correlated to PS1 levels and that down regulating PS1 translates into reduced levels of amyloid β *in vitro* [117,118] providing proof of principle that this might be a feasible therapeutic strategy. Other studies have focused on modulating the expression level or processing of APP either by directly inhibiting APP expression or by influencing expression of APP through manipulation of different other proteins. Directly inhibiting APP expression has been shown to revert phenotypic abnormalities both *in vitro* (endosomal abnormality in Down’s syndrome fibroblasts and rate of apoptosis in cortical and hippocampal neurons from APP transgenic mice) [119,120] and *in vivo* (behavioural phenotype in mice over expressing APP or mutant APP) [56,121], which has encouraged further investigation of RNAi in relation to AD. Influencing APP expression or processing has been explored in different settings. In 2005, Singer and colleagues aimed at targeting BACE1, a protein involved in the processing of APP into amyloid, and by infusion of lentiviral vectors expressing shRNA targeting BACE1 into a transgenic AD mouse model they showed decreased levels of both APP and amyloid β, which was accompanied by alleviation of the behavioural phenotype of the mice [122]. A similar approach has been used by knocking down the DMT1 gene that codes for a protein found to be up regulated in the hippocampus and cortex of transgenic AD mice. These results showed that reducing DMT1 expression was paralleled with a reduction of APP expression and amyloid plaque formation [123]. Finally, since tau pathology is an equally important part of the AD and FTLD pathogenesis, strategies for modulating tau phosphorylation have also been explored. To this end, Piedrahita and co-workers showed that AAV-mediated shRNA knock-down of the cycline dependent kinase 5 (CDK5) in a mice transgenic for PS1, APP and Tau resulted in significant reduction of phosphorylated Tau and reduced levels of neurofibrillary tangles implying the CDK5 might also be a possible therapeutic target in some forms of AD [124].

The avenues of RNAi therapy for AD are numerous possibly because the complex nature of AD pathogenesis. Several genes are known to cause AD, and more genes are probable in the future. In the strategies outlined above, some aim to target the mutant forms of the AD causing genes whereas others aim to target unrelated genes that modulate AD pathogenesis. Since the different disease mechanisms are difficult to dissect completely and since they converge in common pathways, making a strict distinction between therapies aimed at monogenic or non-monogenic forms of AD and FTLD is difficult, which is why they are all presented under the monogenic disorders. However, from a therapeutic point of view this might be advantageous, since one form of therapy might be useful for disease caused by more than one specific mutation and possibly, for disease of unknown origin. Which one of the approaches that is the most promising is hard to tell and it will be interesting to follow the field of RNAi therapy for AD and FTLD in the future, and the wide array of approaches already explored primes the optimism that a therapy will emerge eventually.

### 4.2. Non-Monogenic Disorders

For the polygenic or idiopathic disorders in which the genetic origin of the disorders is complex and often merely unknown, pin pointing a single target for knock-down that will prevent disease progression is most likely impossible. However, although not stopping the disease progression, antisense therapy might be useful for modulating symptoms thereby providing a clinical benefit in such non-monogenic disorders. One example of such a therapeutic strategy has been pursued for PD.

#### 4.2.1. Parkinson’s Disease

**Figure 2:**
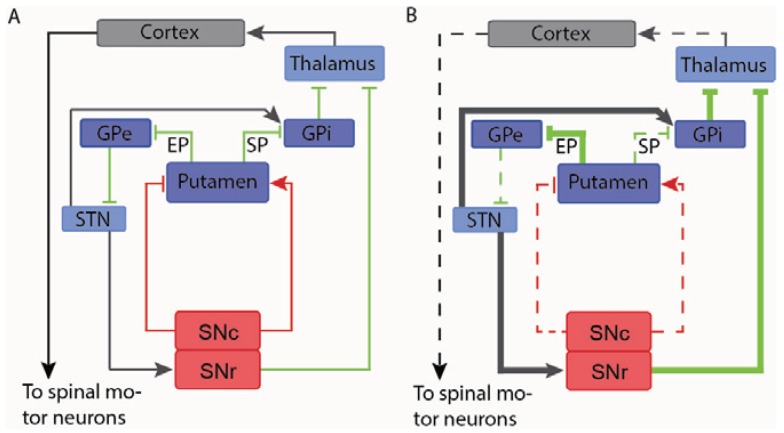
Schematic drawing of the nigrostrital circuitry in the normal brain (**A**) and the parkinsonian brain (**B**). Red, green and gray lines indicate dopaminergic neurons, GABAergic neurons and glutamatergic neurons, respectively. The thin lines indicate normal signal transmission, whereas thick lines and dashed lines indicate enhanced and attenuated signal transmission, respectively. Dopamine depletion in the Putamen (caused by death of the dopaminergic neurons of Substantia nigra) leads to decreased stimulation of the Substance P positive (SP) GABAergic neurons (the direct pathway) that usually receive input from the Substantia nigra through the stimulatory D_1_-receptor [143,144,145,151]. This leads to decreased inhibition of the GABAergic projection neurons of the Globus Palidus interna (GPi). Therefore, the glutamatergic neurons of the Thalamus are inhibited more strongly, leading to decreased stimulation of neurons in the motor cortex [143,144,145,151]. This results in the hypokinetic symptoms characteristic for PD. The decreased dopaminergic input to the Enkephalin positive (EP) GABAergic neurons (the indirect pathway) that usually receive input from the Substantia nigra through the inhibitory D_2_-receptors leads to increased activity of these neurons, which in turn reduces the inhibiting stimulus to the glutamatergic neurons of the Subthalamic nucleus (STN) [143,144,145,151]. Again this leads to increased inhibitory input to the thalamic neurons, which exacerbate the hypokinesia.

Abbreviations: GPe—globus palidus externa, GPi—globus pallidus interna, SNc—substantia nigra pars compacta, SNr—substantia nigra pars reticulate.

In PD the selective degeneration of dopaminergic neurons of substantia nigra results in several changes of the neural circuitry controlling motor function. The dopaminergic neurons innervate two different GABAeregic neuronal populations in the Putamen through D_1_ and D_2_ receptors, respectively. Since the D_1_ receptor is stimulatory whereas the D_2_ receptor is inhibitory, the decreased dopaminergic innervation results in decreased activity of one population (the Substance P positive neurons) and increased activity of the other (the Enkephalin positive neurons). Through a complex neural circuitry, both of these events lead to the reduction of the glutamatergic Thalamic innervations of neurons in the motor cortex resulting in the hypokinetic features that characterize PD (Figure 2) [143,144]. Furthermore, the enkephalin positive GABAergic neurons in the Putamen show up-regulated GABA production due to transcriptional induction of the GABA producing enzyme GAD67 [145], which exacerbate the disturbed neuronal transmission of this pathway [146,147]. This elevated GABA production has furthermore been correlated to motor symptoms [143]. Reversing the pathological increase in GAD67 might then be beneficial to the symptoms of PD. This has been shown by Hovarth and colleagues, who showed that injection of a lentiviral vector expressing shRNAs or artificial miRNA targeted against GAD67 restores normal GABA levels in the Striatum, and that this normalized GABA level is accompanied by the reversal of the pathological increase in neuronal activity that comes with nigrostriatal denervation [111,148]. However, knocking down the GABA producing enzyme in both the Substance P and Enkephalin positive neuronal populations may result in decreased efficacy of treatment or even in side effects, since the reduced GABAergic activity of the Substance P positive population caused by the nigrostriatal denervation will be exacerbated by knock down of the GABA producing enzymes in this particular neuronal population. Hence, specific expression of shRNAs or artificial miRNAs in the Enkephalin positive population might convey a targeted and more precise manipulation of the nigrostriatal system in PD. Such cell specific knock-down in the brain has been achieved [40] and the Enkephalin promoter has been shown to be up regulated upon nigrostriatal dopamine depletion [149,150], suggesting that knock down of GAD67 driven by the Enkephalin promoter might be a feasible therapeutic strategy in PD.

#### 4.2.2. Multiple Sclerosis

Multiple sclerosis (MS) is an autoimmune demyelinating disease affecting the central nervous system. The cause of the disease is unknown, although some genetic polymorphisms have been found to increase the risk. The loss of myelin surrounding axons of the brain and the spinal cord results in various neurological symptoms often involving both physical and mental symptoms, and today no effective treatment to stop the disease exists [125]. However, several compounds inhibiting the immune system have been approved for treating MS, and recently a similar approach of modulating the immune system to treat MS has been explored by Yan and colleagues [152]. In a mouse model of MS they showed that by knocking-down Act1 (a transcription factor involved in mediating interleukin-17 signaling) specifically in astrocytes of the brain an immune suppressive effect in the brain could be achieved and the demyelinating phenotype of the mouse model could be partially halted or prevented [152]. Importantly, by local administration of a viral vector using an astrocytic specific promoter to drive expression of their artificial miRNA targeting Act1, they could avoid peripheral effects of their immune modulating treatment, which is a clear advantage compared to existing treatment regimes.

#### 4.2.3. Prion Disease

Prion disease such as Creutzfeldt-Jakob disease is a fatal and rapidly progressive neurodegenerative disorder characterized by the accumulation of an infectious and protease resistant form PrP(Sc) of the cellular Prion protein [PrP(C)]. It is believed to be caused by an induced conversion of normally folded PrP(C) into the mis-folded form, PrP(Sc). Pathologically this can be observed as amyloid aggregates and the brain tissue adopt a spongiform structure due to tissue damage and degeneration. According to the hypothesis of prion disease being caused by conversion of PrP(C) into PrP(Sc) it should be possible to avoid disease by removing the natural pool of PrP(C) protein. This has been shown by Bueler and colleagues, who showed that mice deficient of PrP(C) have normal development and behavior, but that they are resistant to prion disease [126,153]. Recently, these pioneer studies have been followed up by a study utilizing lentivirus vector mediated RNAi to reduce the level of PrP(C) and thereby slow the progression of prion disease in mice [154]. However, although promising, the knock-down of PrP(C) was achieved using chimeric mice derived from embryonic stem cells transduced with the PrP(C)-targeting vector in order to obtain knock-down almost globally in the brain. Chimeras that carried the PrP(C) targeting construct in a sufficient percentage of the brain showed resistance to inoculation with PrP(Sc) [154] proving that suppression of PrP(C) expression might be a feasible way to treat prion disease, although global expression of shRNA in the brain is far from reality in the clinic.

## 5. Conclusions

Although preclinical studies using RNAi for neurodegenerative disorders are numerous, clinical trials are still very limited. Most clinical studies utilizing RNAi are aimed at the treatment of cancers and viral infections, and for the neurodegenerative disorders the studies initiated so far mostly aim to elucidate the tolerability of small antisense oligos in humans [4] rather than siRNAs. This is likely due to the practical problems still related to the delivery and stability of RNAi molecules compared to the antisense oligos. However, although the use of RNAi for treatment of neurodegeneration has not reached the clinic yet it is still one of the most powerful techniques available to modulate gene expression. The preclinical progress reviewed here provides hope that RNAi therapeutics for neurodegenerative disorders will eventually become reality, not only in disorders with known genetic origin but also in disorders of unknown or multi factorial origin. By further improvement of delivery techniques in addition to more studies on allele specificity and prevention of off-targeting, RNAi holds great promise for therapeutic application—not least in the central nervous system.
